# Do Sex, Age, and Marital Status Influence the Motivations of Amateur Marathon Runners? The Poznan Marathon Case Study

**DOI:** 10.3389/fpsyg.2020.02151

**Published:** 2020-08-31

**Authors:** Patxi León-Guereño, Miguel Angel Tapia-Serrano, Arkaitz Castañeda-Babarro, Ewa Malchrowicz-Mośko

**Affiliations:** ^1^Health, Physical Activity and Sports Science Laboratory, Department of Physical Activity and Sports, Faculty of Psychology and Education, University of Deusto, Bilbao, Spain; ^2^Department of Didactics of Musical, Plastic and Body Expression, Faculty of Teacher Training, University of Extremadura, Cáceres, Spain; ^3^Faculty of Sport Sciences, Eugeniusz Piasecki University of Physical Education, Poznań, Poland

**Keywords:** running, marathoners, motivation, age, sex difference, marital status

## Abstract

The purpose of this research was to describe reasons for participating in a marathon and their association with marital status, age, and sex. Four hundred and ninety-three runners in total, 144 of whom were women and 349 of whom were men, completed the Polish version of the Motivations of Marathoners Scale (MOMS), containing nine dimensions, which was released through an online survey at the Poznan Marathon in Poland (2019). Athletes’ age (ranges 19–25, 26–35, 36–50, 51–70 years) and marital status (single, married, divorced) were asked of the participants. The results showed that statistical significant associations were found between athletes’ motivational aspects and athletes’ sex and age. To this end, three MOMS dimensions were statistically associated with athletes’ sex, a further three dimensions were different age-wise, and, lastly, marital status did not show differences in any of the dimensions. Moreover, statistical differences were not found in the multivariate analysis comparing marital status, sex, and age range according to MOMS. Recreational runners’ reasons for participating in a marathon are different depending on certain sociodemographic variables; therefore, these characteristics should be considered when addressing different athletes in order to provide them with the most suitable information for taking part in such events.

## Introduction

Recreational running has become one of the most common physical activities worldwide, and the number of running events has increased proportionally (Breedveld et al., 2015; Hallmann et al., 2015; Dallinga et al., 2019; Kozlovskaia et al., 2019), as has the number of marathons hosted by different cities in the world (Hulteen et al., 2017). The latter gather thousands of people with different levels of skill who are keen to run such a distance (Buning and Walker, 2016). Due to this popularity, a wide-ranging approach has analyzed these mass sports events, in an attempt to describe, among other aspects: the physical health benefits of running (Hulteen et al., 2017; Oja et al., 2017; Mujika-Alberdi et al., 2018; Kozlovskaia et al., 2019); psychological benefits and mental change (Mazyarkin et al., 2019), finding, for instance, that marathon runners’ mental health was better than that of non-athletes (Raglin, 2007; Boudreau and Giorgi, 2010); endurance running performance-related research (Ferrer et al., 2015); social, tourism, and leisure-related research (Shipway and Jones, 2007; Waśkowski, 2011; Nowak and Chalimoniuk-Nowak, 2015; Summers et al., 2016; Malchrowicz-Mośko and Rozmiarek, 2018; Malchrowicz-Mośko et al., 2019), coaching-related research (Malchrowicz-Mośko and Rozmiarek, 2018), and research involving analyzing psychosocial factors related to marathon running (Summers et al., 2016); and psychological motivational characteristics of amateur or recreational runners (Larumbe et al., 2009; Hammer and Podlog, 2016).

Since running events have increased significantly in the last few decades, organizers have started trying to understand the reasons why athletes would take part in different sports events and to define the reasons why a person decides to participate in such events (Scheerder et al., 2015). A distinction is thus drawn between certain groups with specific characteristics that need to be taken into account when organizing an endurance event (Buning and Walker, 2016). In this regard, a great body of literature has tried to describe the reasons that have led athletes to take part in different sports endurance events, e.g., triathlons (Croft et al., 1999; Wicker and Weimar, 2012; López-Fernández et al., 2014; Myburgh et al., 2014) and cycling events (Lachausse, 2006; Heesch et al., 2012; Malchrowicz-Mośko et al., 2019). Apart from the previously cited sporting contexts, the Motivations of Marathoners Scale (MOMS), developed by Masters et al. (1993), has been used in different running contexts, such as adventure races (Doppelmayr and Molkenthin, 2004), a 5 km running event (Bell and Stephenson, 2014), half marathons (Bell and Stephenson, 2014; Malchrowicz-Mośko et al., 2018), and ultramarathons (Doppelmayr and Molkenthin, 2004; Frick, 2011; Malchrowicz-Mośko and Rozmiarek, 2018; Waśkiewicz et al., 2019a), and for trying to distinguish athletes’ reasons for participation depending on the distance, comparing half marathon, full marathon, and ultramarathon runners’ reasons for participation (Hanson et al., 2015). Other variables such as the type of event, traditional sports events vs. non-traditional sports events (Buning and Walker, 2016), cause-related vs. non-cause-related endurance events (Rundio et al., 2014), first-time marathoners’ motivations, and pre-race dropout reasons (Havenar and Lochbaum, 2007) have been analyzed using the MOMS scale, in order to distinguish and understand what drives athletes to participate in those events. Along these lines, some research has tried to cluster athletes according to their motivational profiles (Ogles and Masters, 2007; Parra-Camacho et al., 2019) or according to their family context, for instance (Goodsell et al., 2013), in an attempt to establish different target groups that may be considered when organizing a sports endurance event.

Athletes’ motivation is the psychological aspect that has been analyzed most, since it helps to explain their participation in a marathon (Ruiz-Juan and Zarauz Sancho, 2014). Several studies have focused on the motivation of recreational runners due to the importance they place on participating in such events (León-Guereño et al., 2020). Many of them have been conducted on the basis of self-determination theory (Deci and Ryan, 2000), whereby athletes’ motivation is divided into two main dimensions, namely, intrinsic and extrinsic motivation (Ruiz-Juan and Zarauz Sancho, 2014). Nevertheless, the creation of MOMS (Masters et al., 1993) was one major development, showing a multidimensional questionnaire designed specifically to assess marathoners’ motives. MOMS, as its name “Motivations of Marathoners Scale” suggests, was created specifically in order to measure marathon athletes’ reasons for participation, and therefore, athletes’ motivations have been analyzed in many marathons from within different social contexts, e.g., in Greece (Nikolaidis et al., 2019), Poland (Malchrowicz-Mośko et al., 2019; Waśkiewicz et al., 2019a), Spain (Ruiz-Juan and Zarauz Sancho, 2014; Parra-Camacho et al., 2019), and especially in the United States (Ogles and Masters, 2003; Rundio et al., 2014; Hanson et al., 2015; Buning and Walker, 2016), the context in which the scale was initially created. The variables associated with different reasons for participating have also been diverse, with the following being the most analyzed variables: runners’ sex (Waśkowski, 2011; Summers et al., 2016; Malchrowicz-Mośko and Rozmiarek, 2018; Malchrowicz-Mośko and Poczta, 2019) and runners’ age (Ogles and Masters, 2003; Reed and Gibbs, 2016; Poczta et al., 2018; Nikolaidis et al., 2019). This constitutes an attempt to describe whether the reasons for a person taking part in an endurance event depend on athletes’ sex and age. Other variables such as years’ experience running (Ogles and Masters, 2003; Malchrowicz-Mośko et al., 2020) or training experience (Waśkiewicz et al., 2019b) and athletes’ performance (Ferrer et al., 2015; Nikolaidis et al., 2019) have also been associated with athletes’ motivations.

Nevertheless, marital status has yet to be analyzed in relation to marathon athletes’ participation motives. Taking into account how peoples’ lives can change depending on their marital status, and the changes that may take place from being single to being married, and also from this latter marital status to being divorced, it would seem to be an interesting variable when analyzing athletes’ participation motives in a sporting event. This may be even more relevant when the sports event in question is a marathon, as this is a very demanding race, with a heavy load or training sessions that need to be completed. Goodsell et al. (2013), in an American sample, concluded that family context was an important influence on peoples’ motivation behind running and that it should be taken into account by researchers and those who wish to encourage long-term engagement in active leisure.

As regards the Polish social context, mass sports events and the ideology of healthism have been developing in the last few years, in view of how Poles’ physical activity has in turn increased over the last two decades. Socio-cultural and economic aspects have influenced this growth of physical activity, with Poles now being better educated, being wealthier, and having more free time for leisure, having seen their quality of life increase (Malchrowicz-Mośko and Poczta, 2019). Within this context, Poles had taken Western countries’ lifestyles on board, and reasons for taking part in sports events such as marathons have increased exponentially. Thus, it is especially interesting to understand this construct, since a huge social change took place within a quite-short period, and athletes’ motivation has also changed too.

Reviewing the literature on the subject showed that most previous research has focused on other countries (Ogles and Masters, 1995, 2003; Rundio et al., 2014; Nikolaidis et al., 2019) rather than specifically on the Polish social context, even though marathoners’ motivational characteristics were analyzed in Poland over 10 years ago (Waśkiewicz et al., 2019b). With this research, we attempt to provide up-to-date information about athletes’ reasons for participating in marathons. On the other hand, marital status has yet to be suitably addressed, this being an innovative perspective put forward by this research. Therefore, in this research, the main aim will be to show why amateur athletes take part in marathons in Poland, and to this end, runners’ motivations will be analyzed and associated with participants’ sex, age, and marital status – this last mentioned factor, as we have already mentioned, has tended to be underestimated in literature. In line with this objective, the main hypothesis of this research is that amateur athletes’ motivational aspects differ according to their age, sex and, marital status.

## Materials and Methods

### Participants and Study Design

Approval was obtained from the Ethics Committee of the University of Deusto, Spain, and the study was consistent with the Helsinki declaration of 2013. Participants were treated ethically under the American Psychological Association code of ethics regarding consent, anonymity, and responses. It is a cross-sectional study, whose total sample comprised 493 participants in the Poznan Marathon; both females (*n* = 144) and males (*n* = 349) filled out the questionnaire. All of them provided written informed consent for participation in the survey, with those athletes who did not ultimately participate in the event being excluded from the study. Participants were recruited via intentioned selection. Individuals took part in the 20th Poznan Marathon in Poland in October 2019. The Poznan Marathon is one of the biggest marathons in Poland and, in fact, one of the biggest in central Europe. 2019 was the 20th year the competition was held. For the first 10 years it was organized, it was the premier running event in Poland, and 6,092 marathoners completed the Poznan Marathon in 2019.

### Measurements

#### Sociodemographic State

Participants, as shown in Table 1, were asked about sex (male, female), age range (ranges <18, 19–25, 26–35, 36–50, 51–70 years), and marital status, to determine the most accepted status (single, married, divorced).

**TABLE 1:** Column profile according to athletes’ age ranges and sex.

	**<18**		**19–25**		**26–35**		**36–50**		**51–70**		**Total**	
**Gender**	***n***	***%***	***n***	***%***	***n***	***%***	***n***	***%***	***n***	***%***	***n***	***%***
Male	2	40	26	57	115	69	185	73	21	88	349	71
Female	3	60	20	43	51	31	67	27	3	12	144	29

#### Athletes’ Motivation

The Polish version of the multidimensional MOMS scale was used (Dybała, 2013), developed initially by Masters et al. (1993). Athletes’ motivation was measured via 56 items or reasons for participating in a marathon, organized using a seven-point Likert scale, with the highest score being 7 and the least-valued motive rated 1. This scale shows nine dimensions that authors divided into four main broader groups of motive: (1) psychological motives, involving self-esteem, psychological coping, and life meaning; (2) achievement-related motives, including personal goal achievement and competition; (3) social motives, showing recognition and affiliation motives; and (4) physical health motives, including general health orientation and weight concern (Masters et al., 1993).

### Procedure

The online survey was set up using Google Docs technology (Šmigelskas et al., 2019). Athletes were contacted via the Internet the same weekend that the 20th PKO Poznan Marathon was taking place in October 2019 and were provided with detailed information about the research by the organizers. Previously, the event organizer was suitably informed about this study, which they passed on to all the participants in the marathon.

### Data Analysis

The statistical analysis was carried out by R Core Team in this quantitative analysis. A Principal Component Analysis (PCA) variable graph was calculated to show an outline of the association and direction of the nine MOMS dimensions (Shahid et al., 2016), thus summarizing the information obtained from the correlation matrix (Supplementary Table A1) and giving an overall view of the correlations among athletes’ different reasons for taking part in a marathon. One-way ANOVA was used in order to analyze the association between athletes’ reasons for participating and the three independent variables selected for this research: age, sex, and marital status. Normality in terms of distribution was verified by the Shapiro–Wilk test, and the assumption of homogeneity of variance was ascertained using Levene’s test. Multivariate analysis of variance was applied to ascertain the importance of marital status (single, married, divorced), age range (<18, 19–25, 26–35, 36–50, 51–70), and sex (female, male) differences in terms of MOMS variables. Multiple *post-hoc* comparisons with Bonferroni corrections and eta square (η^2^) were also used, the latter being a multivariate measure of effect size. The results were considered statistically significant when *p* ≤ 0.05.

## Results

**FIGURE 1:**
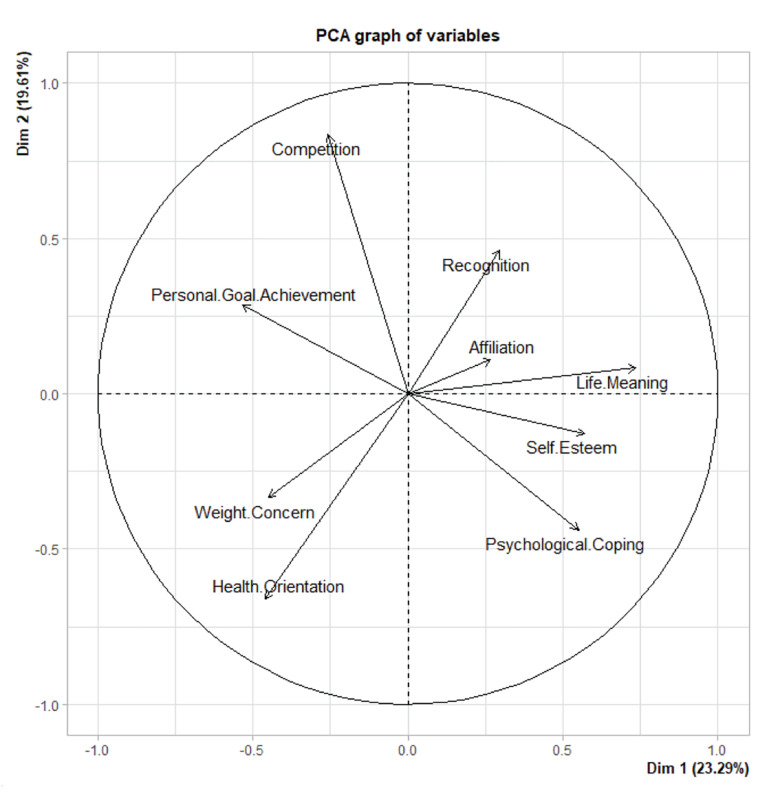
Principal Component Analysis (PCA) graph of variables, showing data for the nine Motivations of Marathoners Scale (MOMS) dimensions.

The results obtained from this research attempted to describe the reasons for participation by amateur athletes and their association with runners’ marital status, sex, and age. Figure 1 – the variables graph (PCA) – showed an outline of the association and direction of the nine dimensions or reasons for participation on the part of marathon runners. The horizontal dimension showed 23.3% of the variation, and the vertical dimension, 19.6%, thus totaling 42.9% of the total variation. Figure 1 presents each motivational dimension of athletes represented by an arrow, thus showing the motives and direction taken by participants, summarized in two axes, enabling us to gain an insight into participants’ motivation. Horizontal and vertical dimensions show two groups referring to dimension, displaying the main motives and direction taken by those participating in a marathon.

The horizontal or main dimension showed opposing directions in terms of reasons for participation, with health orientation, weight concern, personal goal achievement, and competition in one direction and psychological coping, life meaning, and self-esteem in the other direction. In the vertical dimension, Figure 1 shows how competition and recognition are to be found on the upper part of the graph, while conversely, health reasons for participating are on the lower part of the graph. The opposing directions of personal goal achievement and affiliation can be observed on the upper part of the graph in Figure 1.

Table 2 shows that personal goal achievement (*p* < 0.05), competition, and psychological coping (*p* < 0.001) evidenced statistical differences between male and female runners, showing small to medium effect size values. Conversely, the rest of the MOMS dimensions did not show any significant differences in terms of sex.

**TABLE 2:** One-way ANOVA of nine Motivations of Marathoners Scale (MOMS) dimensions according to sex.

**MOMS**	**Women**		**Men**		***F***	***p***	***d***
	***M***	***SD***	***M***	***SD***			
Health orientation	5.50	1.24	5.38	1.32	0.986	0.321	0.03
Weight concern	4.10	1.73	4.25	1.61	0.832	0.362	0.14
Personal goal achievement	5.16	1.34	4.78	1.43	8.052	0.005	0.26
Competition	3.18	1.51	2.59	1.43	16.364	0.000	0.41
Recognition	3.07	1.35	2.9	1.25	1.598	0.207	0.32
Affiliation	3.49	1.65	3.56	1.52	0.207	0.650	0.25
Psychological coping	4.13	1.34	3.78	1.45	15.528	0.000	0.20
Life meaning	3.78	1.46	3.95	1.37	1.464	0.227	0.09
Self-esteem	4.64	1.39	4.91	1.38	3.734	0.054	0.17

Table 3 shows statistical differences according to age ranges in three MOMS dimensions: health orientation (*p* < 0.001), with a medium to large effect size, and affiliation (*p* < 0.05) and self-esteem (*p* < 0.05), with small to medium effect size. Conversely, the rest of the dimensions did not show any significant differences according to age.

**TABLE 3:** One-way ANOVA of nine MOMS dimensions in terms of age.

**MOMS**	**≤18 years *n* = 5**		**19–25 years *n* = 46**		**26–35 years *n* = 166**		**36–50 years *n* = 252**		**51–70 years *n* = 24**		***F***	***p***	***d***
	***M***	***SD***	***M***	***SD***	***M***	***SD***	***M***	***SD***	***M***	***SD***			
Health orientation	4.57	2.44	4.99^a–b^	1.39	5.27^a–b^	1.23	5.63^a^	1.21	6.16^b^	0.99	6.444	0.000	0.03
Weight concern	3.40	1.99	4.11	1.95	3.94	1.69	4.32	1.63	3.90	1.74	1.601	0.173	0.14
Personal goal achievement	4.60	1.80	5.36	1.27	5.22	1.36	4.90	1.37	4.92	1.47	2.238	0.064	0.26
Competition	2.50	1.78	3.04	1.47	3.16	1.49	2.93	1.52	2.86	1.42	0.789	0.532	0.41
Recognition	2.80	2.01	3.14	1.30	3.14	1.38	2.89	1.26	3.25	1.32	1.253	0.287	0.32
Affiliation	3.30	2.23	3.42	1.63	3.28^a^	1.65	3.60	1.54	4.38^a^	1.70	2.849	0.023	0.25
Psychological coping	4.16	1.83	4.58	1.21	4.34	1.37	4.14	1.37	4.46	1.18	1.444	0.218	0.20
Life meaning	3.83	2.13	3.93^a^	1.46	3.89	1.45	3.76	1.39	4.34	1.45	0.981	0.418	0.09
Self-esteem	4.53	2.23	5.21	1.12	4.72	1.42	4.59^a^	1.36	5.18	1.56	2.745	0.028	0.17

*Between-group comparisons are shown in Table 3 with different superscripts (a, b). One mean is significantly different from another mean if they have different superscripts.*

Table 4 did not show significant differences in terms of the nine MOMS dimensions (all, *p* > 0.05). In addition, the effect size value was small to medium in terms of the nine MOMS dimensions according to marital status.

**TABLE 4:** One-way ANOVA of nine MOMS dimensions’ association with athletes’ marital status.

**MOMS**	**Single *n* = 108**		**Married *n* = 355**		**Divorced *n* = 30**		***F***	***p***	***d***
	***M***	***SD***	***M***	***SD***	***M***	***SD***			
Health orientation	5.30	1.43	5.49	1.23	5.69	1.05	1.458	0.234	0.03
Weight concern	4.36	1.72	4.07	1.39	4.25	1.63	1.274	0.281	0.14
Personal goal achievement	5.09	1.39	5.06	1.35	4.74	1.61	0.806	0.447	0.26
Competition	3.08	1.61	3.01	1.47	2.66	1.43	0.941	0.391	0.41
Recognition	3.17	1.38	2.99	1.28	2.76	1.45	1.402	0.247	0.32
Affiliation	3.56	1.69	3.50	1.57	3.50	1.84	0.060	0.942	0.25
Psychological coping	4.39	1.30	4.23	1.37	4.19	1.41	0.654	0.520	0.20
Life meaning	3.99	1.44	3.77	1.41	3.90	1.58	1.010	0.365	0.09
Self-esteem	4.84	1.38	4.69	1.39	4.70	1.46	0.506	0.603	0.17

Table 5 shows the comparison between motivations of men and women based on their marital status according to the nine MOMS dimensions. No statistically significant differences were found in any of the dimensions (all, *p* < 0.05).

**TABLE 5:** Multivariate analysis comparing marital status and age range according to MOMS dimensions: comparison between women and men.

**MOMS**		**Male**										**Female**										**η^2^**	***F***	***p***
		**<18**		**19–25**		**26–35**		**36–50**		**51–70**		**<18**		**19–25**		**26–35**		**36–50**		**51–70**				
		**M**	**SE**	**M**	**SE**	**M**	**SE**	**M**	**SE**	**M**	**SE**	**M**	**SE**	**M**	**SE**	**M**	**SE**	**M**	**SE**	**M**	**SE**			
Health orientation	Single	3.75	0.88	4.94	0.33	5.41	0.20	5.40	0.28	6.83	0.88	5.06	0.72	4.97	0.37	4.88	0.37	6.45	0.47	–	–	0.010	1.149	0.333
	Married	–	–	4.67	0.36	5.59	0.15	5.59	0.10	6.22	0.32	–	–	5.50	0.41	4.94	0.20	5.69	0.17	5.00	0.88			
	Divorced	–	–	5.38	–	5.69	0.62	5.69	0.34	6.08	0.62	–	–	–	–	4.66	0.88	5.83	0.51	6.50	1.24			
Weight concern	Single	1.88	1.18	4.36	0.45	4.33	0.27	4.26	0.38	5.75	1.18	4.42	0.97	5.05	0.51	3.95	0.51	4.61	0.63			0.007	2.372	0.497
	Married	–	–	2.79	0.48	3.80	0.20	4.24	0.14	3.85	0.43	–	–	4.33	0.56	3.87	0.27	4.49	0.23	1.37	1.18			
	Divorced	–	–	–	–	3.19	0.84	4.63	0.46	4.50	0.84	–	–	–	–	4.75	1.18	3.92	0.68	3.50	1.68			
Personal goal achievement	Single	3.58	0.95	5.19	0.36	5.27	0.22	5.21	0.31	5.33	0.95	5.28	0.78	5.12	0.41	4.62	0.41	4.50	0.51	–	–	0.003	0.678	0.828
	Married	–	–	5.68	0.39	5.41	0.16	5.07	0.11	4.73	0.35	–	–	5.50	0.45	5.16	0.22	4.48	0.18	2.75	0.95			
	Divorced	–	–	–	–	4.41	0.67	4.59	0.37	6.17	0.67	–	–	–	–	3.92	0.95	4.36	0.55	6.33	1.35			
Competition	Single	1.38	1.04	3.02	0.39	3.38	0.24	3.53	0.34	2.75	1.04	3.25	0.85	2.73	0.44	2.68	0.44	2.04	0.56			0.009	2.449	0.344
	Married	–	–	2.98	0.43	3.34	0.17	3.16	0.12	2.88	0.38	–	–	3.56	0.49	2.77	0.24	2.34	0.20	1.37	1.04			
	Divorced	–	–	–	–	3.31	0.74	2.19	0.41	3.87	0.74	–	–	–	–	1.63	1.04	2.92	0.60	1.75	1.48			
Recognition	Single	1.67	0.92	3.09	0.35	3.26	0.21	3.48	0.30	3.83	0.92	3.56	0.75	3.06	0.39	2.97	0.39	2.62	0.49	–	–	0.018	3.548	0.08
	Married	–	–	2.78	0.38	3.15	0.15	2.96	0.11	3.37	0.34	–	–	3.81	0.43	2.99	0.21	2.73	0.18	1.41	0.92			
	Divorced	–	–	–	–	4.25	0.65	1.99	0.36	3.92	0.65	–	–	–	–	2.09	0.92	3.14	0.53	1.33	1.30			
Affiliation	Single	1.59	1.12	3.48	0.42	3.72	0.25	3.74	0.36	6.00	1.12	4.45	0.91	3.56	0.48	2.59	0.48	3.36	0.60			0.013	3.851	0.189
	Married	–	–	2.97	0.46	3.03	0.19	3.56	0.13	4.52	0.41			3.74	0.53	3.47	0.26	3.79	0.22	2.08	1.12			
	Divorced	–	–	–	–	4.17	0.79	2.79	0.44	4.46	0.79					2.25	1.12	4.42	0.65	3.17	1.58			
Psychological coping	Single	3.61	0.95	4.08	0.36	4.51	0.22	4.13	0.31	4.56	0.95	4.52	0.78	5.14	0.41	4.20	0.41	4.33	0.51	–	–	0.005	1.15	0.637
	Married	–	–	4.30	0.39	4.02	0.16	4.02	0.11	4.67	0.35	–	–	5.05	0.45	4.72	0.22	4.52	0.18	3.17	0.95			
	Divorced	–	–	–	–	4.89	0.67	3.71	0.37	4.42	0.67	–	–	–	–	4.94	0.95	4.39	0.55	3.89	1.34			
Life meaning	Single	3.08	1.00	3.51	0.38	4.10	0.23	4.15	0.33	4.86	1.00	4.33	0.82	3.99	0.43	3.91	0.43	3.92	0.54	–	–	0.009	2.034	0.403
	Married	–	–	3.86	0.41	3.66	0.17	3.66	0.11	4.47	0.37	–	–	4.63	0.47	3.83	0.23	3.90	0.19	2.14	1.00			
	Divorced	–	–	–	–	4.40	0.71	3.19	0.39	4.71	0.71	–	–	–	–	3.14	1.00	4.74	0.58	4.29	1.42			
Self–esteem	Single	3.75	0.97	4.78	0.37	4.68	0.22	4.94	0.31	5.75	0.97	5.04	0.79	5.59	0.41	4.76	0.41	4.45	0.52	–	–	0.020	4.45	0.052
	Married	–	–	5.12	0.40	4.62	0.16	4.51	0.11	5.18	0.35	–	–	5.56	0.46	4.87	0.22	4.82	0.19	2.82	0.97			
	Divorced	–	–	–	–	5.84	0.69	3.78	0.38	5.75	0.69	–	–	–	–	3.88	0.97	5.17	0.56	6.63	1.37			

## Discussion

The aim of this research was to describe why athletes decide to take part in a marathon, i.e., runners’ reasons for participation, focusing on some characteristics of participants, such as their age, sex, and marital status, with some of these variables being previously analyzed in other endurance races (Nikolaidis et al., 2019; Waśkiewicz et al., 2019b) and in other social contexts (Buning and Walker, 2016; Malchrowicz-Mośko et al., 2019; Parra-Camacho et al., 2019). The main findings showed that some of the variables analyzed, namely, sex and age, influence the reasons for participation by amateur athletes, while marital status did not evidence any such association. As previous research shows, age and sex have been analyzed in a binomial way, in order to ascertain whether sex–age makes a difference, how far it extends, or in which direction these differences exist in marathon race participants (Reed and Gibbs, 2016; Nikolaidis et al., 2018). One of the most analyzed variables has been athletes’ sex when trying to understand endurance athletes’ reasons for practicing their sport. In this case, this study shows that amateur athletes’ reasons for participating in a marathon in Poland are significantly different in three of the MOMS dimensions according to sex, i.e., male amateur runners’ reasons for participating in a marathon are significantly greater in terms of personal goal achievement (*p* < 0.005) and competition (*p* < 0.001) compared to female runners. At the same time, female amateur runners’ reasons for participation are greater than in men in terms of psychological coping (*p* < 0.001). These results are partially in keeping with Stempień (2014), who found that non-performance-related variables were preferred by Polish lady runners, with results showing that male and female runners’ reasons for participating in a marathon were statistically different. Along the same lines, female marathoners in the classic Athens race showed greater reasons for participation in psychological coping and self-esteem than male runners, although personal goal achievement was found to be a meaningful characteristic in the Greek contexts (Nikolaidis et al., 2019). Conversely, in our research, personal goal achievement was male runners’ key factor in taking part in a marathon, and life meaning was a significant reason for participation on the part of Polish female marathoners. In the USA context, Ogles and Masters (1995) analyzed athletes’ reasons for participation in terms of sex, and the results obtained support our findings, i.e., women and men differ in their reasons for participating in a marathon, showing that weight concern, affiliation, self-esteem, life meaning, and psychological coping were more key factors for women than for men; on the other hand, health orientation, personal goal achievement, competition, and recognition would explain male runners’ participation in marathon events – results that coincide partially with the findings of our research. Within these results, in the Polish context, psychological coping was the motive that showed statistical differences among women, while personal goal achievement and competition were the main reasons for participating among men.

Our results coincide partially with previous research, finding an age effect with regard to the reasons for participating in a marathon, with the motives associated with competition being greater in younger athletes than in older runners (Poczta et al., 2018; Nikolaidis et al., 2019). Likewise, in our research, marathoners’ reasons for participating were different depending on their age, with these results being of greater importance alongside personal goal achievement in younger athletes than in older ones, in line with Poczta et al. (2018) and Nikolaidis et al. (2019). However, our research showed that athletes’ reasons for participation also differ statistically age-wise in health orientation, with more concern about health being shown the older the runners get, i.e., the youngest runners evidenced the lowest scores or reasons for participating in this dimension, while it gains importance as athletes get older. Weight concern was also different according to athletes’ age, with the 36–50 age range of athletes showing the most concern about this dimension. Conversely, personal goal achievement lessened in importance as age rose, and affiliation also showed differences among age ranges, with this dimension being of greater concern as athletes get older. Self-esteem showed statistical differences among age ranges, with a decreasing trend the older athletes get. Therefore, our results are, to a great extent, in line with previous results, showing, in general, that younger athletes focus on results and personal reasons for participating, while older runners focus more on meeting other runners and social reasons or on health-related reasons (Poczta et al., 2018).

Little research has been conducted linking runners’ motivations and family context, although this is a very important issue. Moreover, athletes’ motivations need to be understood beyond psychological aspects, and social factors need to be taken into consideration (Goodsell et al., 2013). For many amateur runners, a marathon is a demanding activity, and while being immersed in it, they enter the running social world and undergo a process involving identity transformation. This process encompasses immersion into a zone that is often outside the partnership of marriage and includes absorption into social networks that are unlimited in time and place and, consequently, may jeopardize the marriage (Shahid et al., 2016) – a reason why marital status could be associated with athletes’ participation motives. Within runners’ social context, marriage, marital status or the birth of a child might have a great influence on athletes’ motivational aspects (Goodsell et al., 2013). Our results showed that marital status, i.e., being married, divorced, or single, was not significant in the case of any of the reasons related to the MOMS dimensions. None of the reasons identified by the MOMS showed significant differences when the interaction between age, marital status, and sex was taken into account. These results are in line with Goodsell et al. (2013), as they did not find a statistically significant relationship between marital status and the intention to run. However, they did find a significant association in athletes to continuing running (Goodsell et al., 2013). Lastly, based on these results, marital status was not found to exert a significant association in any of the motivations expressed by marathon runners, after taking into account factors such as sex or age.

However, these results need to be viewed carefully, since they describe the reasons for participation in a marathon within a specific social context, and the research was carried out using a cross-sectional design that did not allow for any causal inferences among the variables. Moreover, personal variables such as type of job or health status and variables such as the birth of a child or the number of children in the family have not been taken into account within the family context – these are some limitations of the research. In future research, collecting data at different times or moments would provide a wider view of the range of athletes’ reasons for taking part in an endurance event (Nikolaidis et al., 2018). Moreover, affiliations could be taken into account from a cultural standpoint in future research (i.e., religion, race), thus providing more specific information associated with family and cultural context, in order to help understand its relationship with athletes’ reasons for running (Södergren et al., 2008). However, analyzing the variable of marital status can be considered a strength of this research.

According to this study, Polish amateur runners’ reasons for participating in a marathon are different depending on whether the athlete is male or female and on their being younger or older, although marital status did not show any such association. Our results suggest that sporting event organizers, health promotion specialists, and coaches should consider how female runners evidence statistically greater motivation than men in personal and social dimensions such as psychological coping, while men are more motivated with result-oriented dimensions such as personal goal achievement, competition, and recognition, in line with previous research (Waśkiewicz et al., 2019b). It is thus understood from these results that women may gain more psychological benefits from running than men (Reed and Gibbs, 2016).

## Conclusion

In conclusion, this study shows that amateur runners’ sex and age matter to a greater or lesser extent when it comes to their reasons for participation. However, no relationship was found between marital status and athletes’ motivational dimensions. It would therefore be interesting for event organizers to use this information when releasing or promoting such sporting events as a marathon, in order to ensure participants’ continued support for these types of competition by meeting different participants’ needs and reasons for participation.

## Data Availability Statement

All datasets generated for this study are included in the article/Supplementary Material.

## Ethics Statement

The studies involving human participants were reviewed and approved by the Ethics Committee of the University of Deusto, Spain. Written informed consent to participate in this study was provided by the participants’ legal guardian/next of kin.

## Author Contributions

PL-G, EM-M, and MT-S contributed to the conception and design of the study. PL-G and AC-B organized the database, performed the statistical analysis, and wrote the first draft of the manuscript. PL-G, AC-B, and EM-M wrote sections of the manuscript. All authors contributed to manuscript revision and read and approved the submitted version.

## Conflict of Interest

The authors declare that the research was conducted in the absence of any commercial or financial relationships that could be construed as a potential conflict of interest.
