# Supplementary material for: Do Sex, Age, and Marital Status Influence the Motivations of Amateur Marathon Runners? The Poznan Marathon Case Study
Source: Front Psychol. 2020 Aug 31;11:2151. doi: 10.3389/fpsyg.2020.02151 (PMC7488354; doi:10.3389/fpsyg.2020.02151)
Supplement: Supplementary file 2 [file Table_1.docx]

**Table 1.** Correlation Matrix

|  | **Health Orientation Rel.** | **Weight Concern Rel.** | **Personal Goal Achievement Rel.** | **Competition Rel.** | **Recognition Rel.** | **Affiliation Rel.** | **Psychological Coping Rel.** | **Life Meaning Rel.** | **Self Steem Rel.** | |
| --- | --- | --- | --- | --- | --- | --- | --- | --- | --- | --- |
| Health Orientation Rel. | — |  |  |  |  |  |  |  |  | |
| Weight Concern Rel. | 0.082 | — |  |  |  |  |  |  |  | |
| Personal Goal Achievement Rel. | 0.034 | -0.228*** | — |  |  |  |  |  |  | |
| Competition Rel. | -0.367*** | -0.211*** | 0.251*** | — |  |  |  |  |  | |
| Recognition Rel. | -0.423*** | -0.048 | -0.285*** | 0.161*** | — |  |  |  |  | |
| Affiliation Rel. | -0.164*** | -0.176*** | -0.331*** | -0.092* | -0.035 | — |  |  |  | |
| Psychological Coping Rel. | -0.068 | -0.187*** | -0.308 *** | -0.374*** | -0.029 | -0.154*** | — |  |  | |
| Life Meaning Rel. | -0.288*** | -0.420*** | -0.291*** | -0.100* | 0.002 | 0.177*** | 0.200*** | — |  | |
| Self Steem Rel. | -0.222*** | -0.279*** | -0.094* | -0.272*** | 0.033 | -0.173*** | 0.227*** | 0.255*** | — | |
| **Note.** * p < .05, ** p < .01, *** p < .001 | | | | | | | | | |  |
